# Understanding the life experiences of elderly in social isolation from the social systems perspective: using Hong Kong as an illustrating example

**DOI:** 10.3389/fpsyt.2023.1114135

**Published:** 2023-07-05

**Authors:** T. Wing Lo, Gloria Hongyee Chan

**Affiliations:** ^1^Caritas Institute of Higher Education, Tseung Kwan O, New Territories, Hong Kong SAR, China; ^2^Department of Social and Behavioral Sciences, City University of Hong Kong, Kowloon Tong, Hong Kong SAR, China

**Keywords:** elderly, socially isolated, social support, quality of life, life satisfaction

## Abstract

**Aim:**

The elderly in social isolation often referred to as older people who experience social alienation with little social support from their family, peers, and community suffer from a poor quality of life and well-being. Since their life experiences are affected by a range of factors from different levels, this study seeks to investigate their current life situations and experiences from a social systems perspective.

**Methods:**

A qualitative study was conducted to enrich the understanding of their current life situations and experiences and to generate corresponding practice implications. In this study, there were 13 elderly participants in social isolation, which were users of a social service agency in Hong Kong. They took part in a semi-structured individual interview, sharing their life stories about their daily lives, social relationships, and sense of well-being. Qualitative results were analyzed based on these dimensions.

**Results:**

Results showed that the elderly participants in social isolation had a low level of social support and participation in social activities. Their life experiences and situations were affected by multiple levels of factors that were interrelated.

**Conclusion:**

The results support the application of the social systems perspective in investigating the living conditions of the elderly in social isolation. The corresponding practice implications were also discussed.

## 1. Introduction

It is undeniable that the global society is aging (1) and this demographic shift is visible in Hong Kong as well. Aging results in an increase in the dependency ratio (2). According to the Census and Statistics Department (3), the elderly dependency ratio, which is defined as “the number of persons aged 65 and over per 1,000 persons aged between 15 and 64 years,” steadily increased from 177 in 2011 to 218 in 2016 and then to 282 in 2021 in Hong Kong. This implies that an adequate amount of family support, as well as service and welfare support, is required to meet these needs (4). With regard to the welfare system in Hong Kong, which is a predominantly Chinese society, Confucian values are upheld, and children are encouraged to accept the moral obligation of filial piety and take care of their parents (5). However, as society changes, family values have transformed, and nuclear families, “consisting of a married couple with or without unmarried children,” have become the dominant family structure in society [(5, 6), p. 688]. As a result, the elderly are “forced to live alone” [(7): 497], experiencing an “empty nest” [(8): 180] and are barely able to receive immediate, proximal support in case of any need and emergencies. According to the Census and Statistics Department (9), households with the elderly who lived alone contributed to over half (58.3%) of the households with elderly only, which was an alarming share. Living alone may constitute a risk for the elderly as they are prone to physical hazards (e.g., falls), financial burden, and a sense of loneliness, and in turn, experience a very poor quality of life (10, 11). Due to a lack of social support and knowledge of community support (12, 13), these elderly are likely to suffer from aggravated social isolation, which further affects their quality of life. Hence, compared to ordinary elderly people, those experiencing social isolation are even more vulnerable and worthy of concern. Owing to the significance of this issue, particularly in the Chinese context, this study seeks to investigate the elderly in social isolation using a sample from Hong Kong. It is expected to generate meaningful practice implications for improving their quality of life.

In the following, literature related to the elderly in social isolation from various cultural contexts is illustrated to provide the background of this study.

## 2. Literature review

### 2.1. The meaning of social isolation

Social isolation is an objective measure indicating one’s “absence of a support system or having reduced social interactions or relationships with family and friends at an individual level, and with a generally low level of social participation in community life” [(14), p. 49]. While there has not been a consensus with regard to the measurement of social isolation (15), social isolation can be indicated by: (1) living alone or lacking intimacy and attachments with other people [(14), p. 49]; (2) having a social network of a small size (16, 17); (3) the level of diversity in social networks (18); (4) the number of confidants with whom the individual interacts [(14), p. 49]; (5) the frequency of contacts within the individual’s social networks [(14), p. 49]; and (6) the level of “participation in social activities” [(19), p. 210]. Apart from the “structural” and “physical” aspects of social relationships such as the “living arrangement” and “size of the social network” [(19), p. 209], social isolation is also about the functional aspect of social relationships, which is the subjective dimension indicating the quality of the social relationships, such as the perceived instrumental and emotional support received from other people (20) and sense of loneliness or lack of belongingness (21, 22). Applying this definition of social isolation to the context of the elderly, it is noted that the elderly in social isolation can be regarded as those who: (1) experience alienation from other people in terms of family, friends, and people in the community; (2) receive weak social support; and (3) and have a low level of social participation.

### 2.2. The life situations of the elderly in social isolation and the related factors

Elderly people in social isolation suffer from a wide range of problems and challenges. In addition to experiencing financial difficulties (4, 23–25), they are at a greater risk of developing physical and mental health issues (26). More importantly, they have a low level of life satisfaction and a low sense of self-efficacy in changing their life conditions and perceive themselves as useless (27). Faced with adverse living conditions in terms of physical, financial, social, and psychological aspects, the elderly suffer from a low quality of life (28, 29). Reviewing existing literature, there are various reasons and factors contributing to social isolation among the elderly.

#### 2.2.1. Intrapersonal factors

One primary reason for social isolation among the elderly is the loss of social roles, such as retirement and worsening health conditions (4, 30, 31). These affect their financial condition and physical ability to participate in social interactions and social activities, respectively (32). Lack of financial resources is viewed as a reason for being socially isolated (33) because low affordability restricts their engagement in activities involving higher-value consumption, and, in turn, restricts opportunities to meet friends through these activities (34, 35). Due to deteriorated health and physical disabilities, their mobility to step outside, participate in activities (e.g., watching movies) (36, 37), and engage with other people (38) is restricted. This hinders the development and maintenance of social networks, which aggravates social isolation and loneliness (37, 39).

In addition, the elderly’s personalities also contributed to their social isolation (31, 39, 40): they either prefer being alone (41) or display a reluctance to seek help from family, friends, and neighbors, for fear that they would be viewed as a burden to others (36). This not only exists in Western societies but also in Chinese society where the elderly in social isolation displays a sense of self-reliance for fear that they would bother other people (27, 30). As a result, they believe that seeking help is morally wrong; thus, they are reluctant to seek service and welfare support. The low self-image and self-esteem issues faced by the elderly might also reduce their likelihood of admitting the need for help and seeking corresponding assistance, which further increases their alienation from society (30, 31, 40). Thus, elderly people’s sense of loneliness can itself be regarded as a risk factor for social isolation as well since feelings result in a perception of a narrowing of social networks (42).

In addition, the elderly’s abilities constitute their state of social isolation. The lack of knowledge on how to seek assistance affects the level of community resources that they receive (43–45). As stated by the Commission on Poverty (32), these elderly are disengaged from society with little information on resource support due to illiteracy or low levels of education. Apart from this, studies have shown that social competencies contribute to social isolation among the elderly. Low levels of social competence result in poor coping strategies against challenges and adversities, which include denial and avoidance of problems and even withdrawing from society (46–49). This leads to further problems such as financial difficulties, depression, and lack of self-care or personal hygiene (50–52).

#### 2.2.2. Interpersonal factors

One of the interpersonal factors of social isolation among the elderly is the death of spouses and peers (53). Some of them experience alienation or separation from family members and long-term family conflicts (27, 54). Loss of social circles is particularly concerning as social relationships and social support serve as a buffer against stress (55, 56) through the provision of various resources to cope with life events [e.g., illnesses and life transitional events (57)]. In this sense, the elderly with shrinking social networks and social support are more likely to experience a sense of loneliness, isolation, and hopelessness (13, 39, 53).

#### 2.2.3. Social or community factors

Societal factors play a role in contributing to the social isolation of the elderly. One factor is related to access to the welfare support system (58). Pension policies affect the level of income received by the elderly, thus influencing their degree of social participation (58). Such instances also occur in the Chinese context such as in Hong Kong where some of the elderly do not receive social assistance or security because they are not considered eligible applicants (32). As noted by Kühner and Chou (59), the costs and difficulties in the procedures for applying for social security constitute a barrier for the elderly to receive financial assistance from the Government. The level of welfare support received by the elderly appears to have cultural differences. While some of the Western countries (e.g., Norway and Finland) adopt a social-democratic welfare regime characterized by a high level of Government intervention, generous social assistance, and universal provision of services which may help promote social inclusion (60), the welfare regime in Hong Kong is featured by minimum Government intervention and no public pension scheme, and the caretaking responsibility is shouldered by the family (61). As a result, the elderly in Hong Kong experience an inadequate amount of social assistance and community service (59, 62) and are more prone to social isolation.

Another factor is related to the culture. In Western countries (e.g., the United States) where individualism, featured by “independence” and “self-reliance,” is upheld, the elderly prefer “intimacy-at-a-distance” and living apart from their children [(63), p. 245]. Oppositely, in Hong Kong, a Chinese society where the Western value of individualism has emerged (6), familial and neighborhood relationships have become less prominent nowadays (64, 65). This generates differences in values between generations: while the elderly value familism [high level of family support (66)], the younger generation tends less to endorse the Confucian values of filial piety (5). Meanwhile, the Government emphasizes Confucian values and ethics in which children have a moral duty to take care of their parents instead of relying on the welfare system [(5): 734]. As such, it affects the functioning of family support: despite the fact that the elderly are “part of an informal network,” it does not necessarily mean that they are provided “with strong and reliable support” [(5): 734]. It also affects the life satisfaction of the elderly (66), and they have to rely on themselves to support their living. Besides, it is noted that the resilience of the elderly is contributed to by historical life events [e.g., War and Cultural Revolution (67, 68)], asserting that “their lives were much better if compared to when they were young” [(68), p. 1043]. This suggests that the elderly in social isolation in Hong Kong may adopt psychological adaptation to lead their lives.

### 2.3. Insights from the above literature: looking at the phenomenon from the perspective of social systems

The above literature illustrates the life situations of the elderly in social isolation, as well as the underlying factors which are intrapersonal, interpersonal, and societal. This results in an overall poor quality of life in terms of physical, financial, social, and psychological well-being. It is noted that these factors are mutually influential; for example, apart from the shrinking social circles, the adverse physical and financial situations of the elderly discourage their participation in social activities, which further limits their ability to build and maintain social networks and, in turn, affects the amount of social support they receive. While it is widely stated that living alone and social isolation of the elderly contribute to their negative well-being, loneliness, and a lowered quality of life (69–72), the perceived quality of social relationships and social support received appears to play a part in affecting these elderly’s sense of well-being, and it might be influenced by socio-cultural factors. In non-Chinese cultural contexts, living alone does not necessarily bring about a sense of loneliness (73). In contrast, in Chinese contexts where family and social cohesiveness are valued, the elderly who live alone are more likely to experience loneliness (10, 11, 74). This suggests that the elderly in social isolation in Chinese contexts, such as in Hong Kong, are likely to experience a lower sense of well-being and a lower quality of life. The intertwining influence of the multi-level factors suggests the plausibility of using the social systems perspective (75–78) to investigate elders in social isolation.

**Figure 1:**
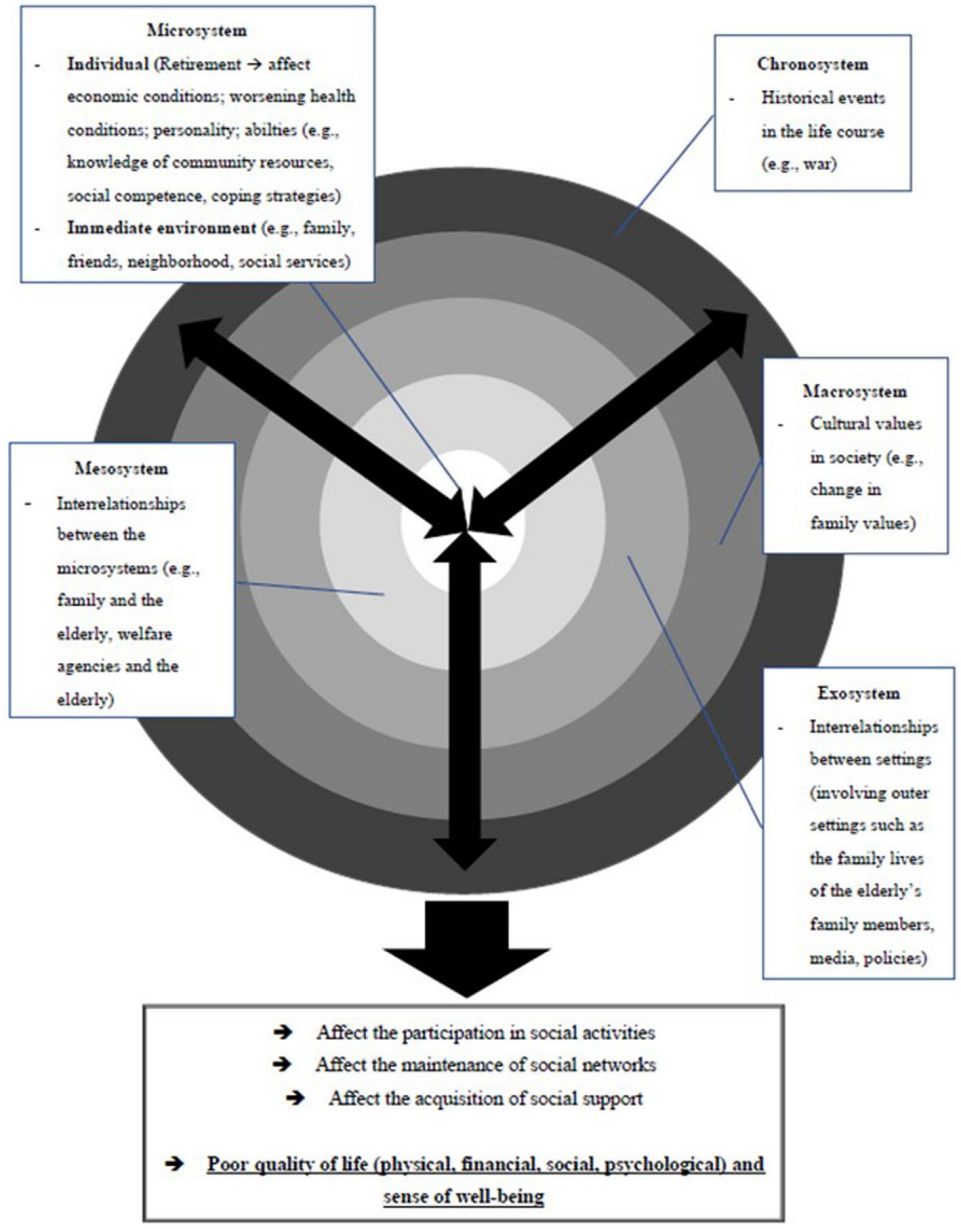
The theoretical framework of this study.

The social systems theory posited that “events in different social units do not occur in isolation but affect one another both directly and indirectly so that changes in one unit or subunit reverberate and impact upon other units” [(79): 36]. In this sense, the functioning of all social systems is relevant to one’s individual life experiences and sense of well-being. Based on Bronfenbrenner’s (75, 76) ecological systems theory of human development, there exist various systems including micro-, meso-, exo-, and macrosystems in which they are reciprocally related (75). The microsystem includes the individual’s personal characteristics (e.g., personality, values, and beliefs) as well as the closest, immediate environment that the individual interacts with [e.g., family, friends, neighborhood, and social services (76)]. The mesosystem consists of the linkages between the microsystems, in which each of them contains the individual (75). The exosystem “encompasses the linkage and processes taking place between two or more settings,” in which at least one of them does not contain the individual “but in which events occur that influence processes within the immediate setting that does contain that person” [e.g., media and policies (76); p. 227]. The macrosystem “consists of the overarching pattern of micro-, meso-, and exosystems characteristic of a given culture, subculture, or other broader social context” [e.g., cultural beliefs (76); p. 228]. The chronosystem “encompasses change or consistency over time not only in the characteristics of the person but also of the environment in which that person lives” [(80), p. 40], such as historical events in life (81). Based on the literature review about the various factors of social isolation of the elderly as illustrated above, Figure 1 presents the theoretical framework, which illustrates these factors from the social systems perspective (see Figure 1).

Despite the existence of literature about the living conditions as well as the related intrapersonal and interpersonal factors of social isolation of the elderly in various cultural contexts, it is noted that the societal factors, which embed the influence of culture, are likely to constitute differences between these elderly in different places. Therefore, it is meaningful to comprehensively investigate their life experiences and conditions from the perspective of social systems. In view of the scarcity of relevant research, particularly in Hong Kong (where research on this group is lacking), this study aims to fill this research gap in order to provide valuable policy and practiceimplications.

### 2.4. Present study

Against this backdrop, this study seeks to investigate the life situations and experiences of the elderly in social isolation from the social systems perspective, using a sample from Hong Kong. Due to the lack of concrete, official definitions of the elderly in social isolation in Hong Kong (82), this study adopts the definition based on existing literature. This includes those: (1) living alone or lacking intimate attachments and frequent contacts with other people including family, friends, and the community; (2) having a small social network, with low diversity and few confidants to interact with; and (3) having a low level of participation in social activities (e.g., leisure, entertainment, and social networking), which cause them to receive little social support and experience sense of loneliness. Simply put, the defining criteria for the elderly participants in this study were related to the quantity and quality of the social relationships perceived by them, which affected the social support they received and the sense of well-being experienced.

## 3. Materials and methods

### 3.1. Participants

In this study, there were 13 elderly participants in social isolation. They were recruited through referrals from elderly centers. Table 1 shows the demographic background of the participants. It is found that over 90% of them were women, and more than 80% were aged 61–80. In addition, their education level was mainly at the primary school level or below (61.5%, *N* = 8). All of them were unemployed; around half of them (53.9%, *N* = 7) had an income of $3,000 or less. This reflects that the elderly in social isolation have a low socio-economic status.

**Table 1:** The demographic background of the participants.

Variables	*N* (%)
**Gender**	
Male	1 (7.7%)
Female	12 (92.3%)
**Age**	
51–60	1 (7.7%)
61–70	4 (30.8%)
71–80	7 (53.9%)
81–90	1 (7.7%)
**Education background**	
Primary or below	8 (61.5%)
Secondary level	4 (30.8%)
University or above	1 (7.7%)
**Employment status**	
Unemployed	13 (100%)
Part-time job	0 (0%)
Full-time job	0 (0%)
**Monthly income**	
$1,000 or below	6 (46.2%)
$1,001–$3,000	1 (7.7%)
$3,001–$5,000	5 (38.5%)
$5,001 or above	1 (7.7%)
Not mentioned	0 (0%)

### 3.2. Data collection procedure

This study adopted qualitative research. An in-person interview with the researcher was arranged for each of the elderly participants in social isolation by the social workers of the elderly centers. They took part in individual interviews from November 2020 to August 2021 where they described their living conditions and experiences in terms of their daily living, social support, sense of well-being, and satisfaction in life. The interviews were conducted either in the service centers or at the participants’ homes. Each interview lasted for 2–3 h and was audiotaped for subsequent transcription of qualitative data as life stories and situations. The interviews were conducted in Chinese in the form of semi-structured interviews, which enabled the researcher to collect in-depth information about the participants’ lives. This is based on the adoption of narrative research, which can be defined as “collecting and analyzing the accounts people tell to describe experiences and offer interpretation” [(83): 179]. It seeks to “unravel the consequential stories of people’s lives as told by them in their own words and worlds” (84). Adopting this methodology, the researcher understood the participants’ narration of their life experiences and how they perceived these experiences from their perspectives (85–87).

### 3.3. Analysis

Thematic analysis was performed to investigate participants’ verbatim accounts in terms of their life experiences and sense of well-being. The following steps were taken: (1) “familiarizing with the data,” (2) “generating initial codes,” (3) “searching for themes,” (4) “reviewing themes,” (5) “defining and naming themes,” and (6) “producing the report” [(88), pp. 87–93]. The analysis was conducted on a theoretically driven basis (88), in which the participants’ life stories were analyzed with themes that showed how different levels of factors (intrapersonal, interpersonal, and societal) intertwined to affect their life experiences and conditions from a social systems perspective. Before the analysis, audio recordings were written in the form of life stories. After studying the life stories, codes were generated from the data, followed by the formation of themes and subthemes relevant to the topic of study. The analysis was cross-checked by two researchers from the research team. In addition, the themes and subthemes, along with the relevant verbatim accounts, were translated by a specialist. Table 2 summarizes the themes, sub-themes, and the corresponding coded qualitative data.

**Table 2:** Themes in the qualitative results.

Themes	Subthemes	Examples of codes
Lack of social activities and its related factors (home-based activities; little leisure and social activities)	Intrapersonal factors	Poor physical conditions; poor financial conditions; other personal factors
	Societal factors	Issues in social services and welfare system, as well as the environment; pandemic
Factors affecting the elderly’s poor social support	Intrapersonal factors	Participants’ own attributes
	Interpersonal factors	Death of spouses; physical distance from family; family conflict; loss of friends owing to life-transitional events and physical distance
Outcomes of the lack of social activities and lack of social support received	Low satisfaction in life	Poor sense of satisfaction; lonely; boring
	Poor sense of self and well-being	Useless; unworthy; unhappy Sense of self and identity attached to family well-being

## 4. Results

### 4.1. The living conditions of the participants

#### 4.1.1. Lack of social activities

All participants’ activities were centered at home (*N* = 13). These were daily routines, including cooking or preparing meals, household chores (e.g., sweeping the floor and washing clothes), watching TV, doing physical exercise at home, and taking rest; some of the participants needed to take care of their spouses who had illnesses or disabilities (e.g., H2, H4, and H6). Although some of the participants would leave home, their activities were around the community and neighborhood nearby (*N* = 8), such as parks for doing exercises (*N* = 5) (e.g., H1: “I get up at around 5 a.m. to 6 a.m., then I go to the park for jogging for 3 h.”) and nearby shops and markets to chat with other people (*N* = 2). Only one participant mentioned that he went to eat dim sum with his children. This shows that the elderly mainly lead home-based lives with little participation in leisure and social activities.

#### 4.1.2. Lack of social support

Participants received poor social support from family, friends, and communities or neighborhoods. Regarding family support, although all the participants had family members, the nature of their family relationships differed. Only three participants received adequate or even good family support (for example, H5 mentioned that his children gave him pocket money; H7 mentioned that “I have good relationships with my children, and my daughter will initiate a meeting with me sometimes.” H8 also mentioned that “I have good relationships with my children; although I do not receive pocket money from them, they will be glad to help me when I am in need”). The majority of them mentioned that they received little family support (*N* = 10). For instance, regarding financial support, only two of them expressed that they received financial support from the family (e.g., H5 mentioned that his children gave him pocket money apart from receiving an old age allowance); participants expressed that they did not receive financial support from their family (e.g., H11: “…I only have the money earned from selling cardboard…after all, I do not have any other sources of income. Even my sons and daughters do not give me any money, not even a few thousand dollars”). Only three participants mentioned that they had peer support, but overall, peer support was weak. A majority of them even mentioned that they had no friends (*N* = 10). Support from the community or neighborhood was even lower. Although some of them might chat with their neighbors, these relationships were not close (H6). There was little contact, with little mutual help and sharing of in-depth issues (H13). For example, H4 mentioned, “even though I have met some people from elderly rehabilitation centers, I seldom see them, and I only greet them without sharing in-depth thoughts and feelings with them… If I encounter any problems, they cannot offer help to me.”

### 4.2. Factors affecting their life experiences

#### 4.2.1. Factors related to their lack of social activities

##### 4.2.1.1. Participants’ deteriorating physical health

Participants’ health conditions constituted an intrapersonal factor affecting their participation in social activities [i.e., the effect of the microsystem (75, 76)]. Only two out of 13 participants showed no major issues in their health (e.g., H5: “…now I consider myself as healthy, without diabetes or other illnesses, only that sometimes I cannot walk smoothly. I think I have not seen the doctor for over 10 years”). A majority of the participants expressed that they had deteriorated physical conditions (*N* = 11), such as diabetes (H1 and H8) and other physical disabilities such as osteoporosis (H10) and chronic pain in the legs and waists (H2, H3, and H4) due to falls (H4: “A long time ago, I fell when I worked in an elderly home. This resulted in problems in my waist.’). Their declining physical abilities affected their engagement in daily life and social activities (*N* = 6). For example, H3 expressed that “As I feel pain in my leg, I need to apply adhesive tape on my leg to reduce it, and this causes difficulty in walking around and doing housework”. H4 also expressed that “My pain in the leg affects my ability to walk around.”

##### 4.2.1.2. Participants’ role as a caretaker

Two participants, who had husbands, expressed that they needed to spend a lot of time taking care of their husbands. For example, H6 expressed that she could not go to any far-off places: “I did not dare to do so [going to any far-off places], as I needed to take care of my husband. I am afraid that he will fall home.” How the participants’ relationship with spouses affects their engagement in social activities demonstrates the role that the mesosystem plays in their social isolation (i.e., the interplay between the microsystems of family and the individual) (75, 76).

##### 4.2.1.3. Participants’ financial conditions

As a result of their old age, participants could not find jobs and had to retire (e.g., H1); thus, they did not receive income. A majority of the participants were recipients of social security (CSSA, old age allowance) (*N* = 11). The amount of social security received per person was approximately $3,000 (e.g., H3, H10, and H12). Only one participant mentioned that she and her husband could get a CSSA of $7,500 (H7). From the other two participants, one mentioned that he earned income from rag-picking (H11: “…I only have the money earned from selling cardboard…after all, I do not have any other sources of income…’), while the other mentioned that he made use of his own savings to meet daily expenses (H8: “I now rely on my savings to get by”). The participants mainly perceived the money received as inadequate (e.g., H11: “The money I have is not enough for me…”) and they were not satisfied with it (e.g., H10: “I’m not quite satisfied with my financial condition”). After paying for the daily expenses (e.g., H10: “As my husband said, our lives were very difficult. We have to pay thousands of dollars for rent and utilities, and I have to go to the doctor…the daily expenses are so expensive…”), they could not afford a higher level of expenses, such as eating the food they wanted (e.g., H7: “Although we have got two sums of money from CSSA, approximately $7,500 a month, I still cannot eat whatever I want very often. Can I afford to eat lobster every day even though its price has dropped to $100 each?”; H10: “…having little money, we can only afford to buy cheaper food and meals”). In addition, owing to their poor financial well-being, their daily activities and consumption were only limited to home-based activities and daily necessities (*N* = 8). For example, H7 expressed that “So even with this money, we still have to use it sparingly to save money for emergencies like buying medicine and seeing the doctor. I rarely go out shopping now, and grocery shopping is already my leisure activity.” While H11 said, “I do not have enough money…I need to wait for a sale when I buy food and ingredients.” This affected their pursuit of personal interests (e.g., H18: “I do not have many interests…but it’s somehow related to the fact that I’m poor, not wanting to spend much money on myself.”) and participation in social activities as well. As H1 mentioned, “I have many interests, but I lack money to participate in them. And because I have no money, I cannot afford to maintain relationships with other people.” In summary, participants’ affordability for social activities was affected by their decreased income, which resulted from retirement and inadequate support from welfare services. This also exhibits that the mesosystem plays a part in affecting their social isolation [i.e., the interplay between the microsystems of work settings and the individual, as well as the welfare agencies and the individual (75, 76)].

##### 4.2.1.4. The barriers to the social service or welfare system

The unfriendliness of the social service or welfare system may be a societal factor affecting the financial conditions of the participants. One participant expressed that although he managed to receive CSSA in the end, the process was very difficult: “I had applied for CSSA before. I felt rejected throughout the process. I was told that I was not eligible. Although I managed to get it (CSSA) at last, I still feel angry when I recall the process” (H1). This supports the existing literature on social services and welfare system issues that constitute obstacles for the elderly (32, 59). While the elderly had to rely on social security to make a living, the welfare system is not applicant-friendly, which results in the needy elderly being barred from the financial support provided at the societal level. The barriers to the social service or welfare system experienced by the participants show the influence of the microsystem on their financial conditions (75, 76).

##### 4.2.1.5. Prevalence of the pandemic

Additionally, the pandemic has also affected the elderly’s engagement in activities. As stated by H3: “…Sometimes I would sit in the nearby convenience stores and chat with the neighbors, but now I seldom do so because of Covid”; also, H4 stated that “I used to do exercises near the river at around 6 a.m., but I do not go out now because of Covid…also, I joined the activities in the community like the feast for the elderly in the past, now I cannot join anymore because of Covid.” This suggests that the pandemic is a factor related to the chronosystem (i.e., a global issue) that affects the occurrence of social isolation among the elderly (80).

#### 4.2.2. Factors related to the poor social support received

##### 4.2.2.1. Participants’ own attributes (intrapersonal factors) which affected their building of social support networks

Participants’ personalities also contributed to social isolation (*N* = 4). For example, H5 mentioned they “prefer being quiet” and H8 said they “do not like to chat with others”; H5, who was afraid of nosy people, believed that “other people cannot help me with my family life and life directions,” thus he chose to be independent and handle things himself.

On the other hand, it was found that participants’ language abilities affected their seeking of social support (*N* = 1). One participant mentioned that he was illiterate: “After all, I am illiterate. How do I dare to ask anything from other people?” (H12). This reflects that the ability of the elderly also affects their ability to build social networks with other people and seek help and support from them.

##### 4.2.2.2. Participants’ death of spouses, with large physical distance from other family members

Among the 13 participants, three lived alone due to the death of spouses (e.g., H1: “I now live alone…in the past, I lived with my husband who was around 20 years older than me; now he passed away”) and six of the participants lived with their spouses only as their children or other family members did not live with them. Among the 13 participants, seven had all their children moved out of home, showing that the participants were affected by the prevalence of nuclear families in Hong Kong (i.e., a societal culture, the macrosystem) (76). They experienced physical distance from other families (*N* = 7): while some of the family members (e.g., children and siblings) lived in other districts in Hong Kong, some of them even lived in the mainland (e.g., H6: “I have two daughters and a son, but they live in the mainland”). This suggests that the elderly often have to count on themselves in their daily lives without instant proximal support from other family members. As H3 expressed, “…my sons ‘have to take care of themselves and cannot help the older ones’. After all, they have to work to ‘raise three people in the family all by themselves’…”; H2 even expressed a sense of an “empty nest” featured by the feeling of grief and loneliness owing to the fact that the children have gotten married, moved away from home, and led their own lives [(8): 180] (H10: “We seldom meet one another…I do not dare to bother them every day…because they have to work and earn a living…”). The work and family lives of the participants’ family members constituted a factor of the exosystem which indirectly affected the participants’ level of social support received and their state of social isolation (76).

##### 4.2.2.3. Poor family relationship or conflict experienced by the participants

Two participants mentioned that they had poor relationships with their family members and even had conflicts with them. For example, (H6: “My children have not given me much money all these years, and I have never told them that I do not have money…I feel that my children will not give me money anyway, whether I have asked them to do so or not. If they were really worried about me, they would ask me whether I had enough money. But they did not.”). H4 even said that “I do not expect them [family members] to support me…I have a poor relationship with my daughter-in-law. After all, she is not my daughter, and my son favors her more.” This reflects that even when the elderly have family members, they merely receive adequate and responsive support from their family to fulfill their needs, which reflects the degree of functioning of the family microsystem experienced by the elderly (75, 76). As such, the elderly participants still have to cope with the issues and challenges in life themselves.

##### 4.2.2.4. Physical distance from friends resulting in a loss of social circle

Two participants mentioned that their friends were lost due to physical distance: H3 expressed that he had fewer friends “because they have moved away, and we no longer maintain contact,” while H7 expressed that “They have moved to different places, so we seldom contact one another.” The participants’ distant relationships with friends reflect the influence of the microsystem on their social isolation (75, 76).

##### 4.2.2.5. Life-transitional events resulting in a loss of social circle

The participants used to have jobs, but after they retired, such connections with colleagues were lost (*N* = 3). For instance, H9 expressed that “I used to have friends who I met at work, but since I no longer work, I no longer maintain contact with them.” This shows that retirement, a change in the microsystem in terms of work, inevitably leads to the loss of one’s social circle (75, 76). For H2, she mentioned the influence of the life events and changes that occurred in her social circle: “…Sometimes I wish to find friends, but their husbands have retired; I cannot ask them to ignore their husbands…I used to have many friends, but now some of my friends have passed away….” While the death of her friends constituted a change in her microsystem, her friends being wives after marriage constituted a factor of the exosystem which indirectly affected her social support received and her social isolation state (76).

#### 4.2.3. Outcomes of the lack of social activities and lack of social support received

##### 4.2.3.1. Low satisfaction in life

Six participants were dissatisfied with their lives. Two of them were not satisfied with the physical, financial, and social conditions (e.g., H2: “I’m not satisfied with the health, not satisfied with the place I live, and not satisfied that I have little money”). The other two were not satisfied due to the lack of leisure or social and meaningful activities (e.g., H3 described his life as “boring” or “not meaningful” because he had no work to do, and his friends had all moved away). The remaining two participants expressed their sense of loneliness and wished to be cared for: H6 said, “I have little to share with my husband, and do not have a close relationship with my children, not to mention other family members…I feel lonely; no one cares about me. I am afraid that nobody knows when I die.” H11 expressed, “My daughter and son have not given me any money, not even a thousand dollars.” This implies that participants particularly wish to be cared for by their family members. Even though they did not enjoy their lives, they believed that they needed to accept their situation. For example, H8 expressed that he could not do anything to improve the situation: “If anyone can give me a job, I would like to do so, like being a domestic helper, but I am too old.” This reflects that the elderly perceive their lives negatively and cannot find meaning in life, and they have a low sense of efficacy in improving their quality of life.

It is noted that they have a sense of independence and self-reliance when dealing with the challenges and hardships that they face. For example, H1 mentioned that “I would strive for happiness myself,” while H13 mentioned “I have nothing to pursue in life… what I can pursue… I’ll withstand (the adversities) when I feel unhappy… no one can help me, not even my daughter.” These findings reflect low expectations of the elderly from life and their psychological adaptation to life issues and challenges. Such resilience and perseverance may be related to their past experiences in life. Most of the participants were Chinese immigrants (*N* = 11); for the rest of the participants, one of them was born in Hong Kong and had the experience of living in the mainland. Some participants who had lived in the mainland described their lives in the past as difficult (*N* = 10; e.g., H2: “In the past, it was difficult to lead a life and even have food, not to mention going to school and study”; H7: “I would say my life in the countryside was ‘miserable’ …at that time, we needed to do physical labor…to lead our lives”); four of the participants even mentioned their experiences of historical events in life (e.g., invasion of China by the Japanese and Cultural Revolution): for example, H1 mentioned that “…and then Cultural Revolution emerge, my father was denounced, but my mother was fine because she was protected by the cadres” [*sic*], while H13 mentioned that “I came to Hong Kong when I was 8 years old…because I could not send money back to Guangzhou…but the water quality there was poor, and my aunt died…”. These are the factors related to the chronosystem (80), which are contributive to their resilience and psychological adaptation during social isolation (e.g., H13: “I used to farm, so I am get used to physical labor…it is a must to work hard” [*sic*]).

##### 4.2.3.2. Poor sense of self and well-being

Although approximately half of the participants remained neutral, without expressing special thoughts and feelings (*N* = 7), the rest of the participants held a negative attitude or perception towards themselves (*N* = 5) than a positive one (*N* = 1). While one participant perceived herself as “tough” and “perseverant” due to her ability to cope with challenges in life (e.g., taking care of the family alone) and negative life events (e.g., death of family members) (H1), the other five of the participants appeared to view themselves negatively. For example, H6 said “I do not have any dreams…because I think I am useless and do not study well,” while H7 felt “ashamed” and “unhappy,” perceiving himself as “useless,” and “not being able to do anything.” “Because I rely on support from the government, such a life I’m leading is not worth honoring.” This reflects that the elderly’s sense of psychological well-being is affected by their living conditions and experiences. Those with low self-images cannot recognize their strengths and unique qualities from what they have encountered in life. Some of them have even internalized the stigma of being a “dependent” recipient of social securities (89), which implies an influence by how the mass media, an exosystem (76), portrays the recipients of social assistance (59). They also have a low sense of self-efficacy and empowerment.

Among the 13 participants, only one mentioned his dream (H2: “learning other languages or dialects for going on trips to other countries”). Five of the participants had no personal dreams, whereas seven of the participants’ dreams and wishes were tied to their families. For example, H6 mentioned that the most important thing to treasure in her life was her husband: “not because we have a good relationship, but think that it’s responsibility to take care of my husband wholeheartedly [*sic*],” while H8 mentioned that, “I do not have any other expectations, except that my children can have a good life.” This reflects their family-based values, and their sense of self and identity is attached to the well-being of other family members.

## 5. Discussion

Our results supported the previous research that the elderly participants in social isolation had a low level of social support and social participation (27, 34, 35, 54). Their life experiences and conditions were affected by a range of interwoven factors of multiple levels (microsystem, mesosystem, exosystem, macrosystem, and chronosystem) (75, 76, 80), which support the application of the social systems perspective in analyzing social isolation of the elderly. Among these factors, it was found that one dominant factor affecting their life experiences is family. Compared to a wider community, participants sought help from their closest family members, aligning with the Chinese traditional values which uphold the importance of family, the people they trust most (90). Despite the existence of family ties among the participants, they merely received adequate instrumental and emotional support from their family; thus, they tend to accept that they had to rely on themselves to solve problems in their daily life, such as spending money sparingly in order to afford the daily expenses as much as possible. This is consistent with the existing literature that the elderly are influenced by the prevalence of nuclear families in Hong Kong (91), in which they can no longer have all their needs “fulfilled within the family” [(6): 687]. However, it is seen that a considerable number of the elderly have dreams and meaning in life that are highly tied to the well-being of their family members (e.g., spouses and children) rather than their own. This shows that their individual well-being is highly determined by their “attachment to the family” [(6): 687]. Hence, their level of satisfaction is highly dependent on their family functioning. When receiving weak family support, they are likely to experience low satisfaction and a poor sense of well-being (66). This suggests that to enhance the quality of life and sense of well-being of the elderly, it is important to reconstruct their lives and sense of self and identity so that they can find multiple pillars in life that help them maintain a sense of life satisfaction and well-being.

Besides, results show that the elderly in social isolation use their psychological adaptation, lowering their expectations in life, to tackle the adversities and challenges in life by themselves. Meanwhile, the life stories of the participants showed that they felt a sense of loneliness, hoping to be reached out to and cared for by other people. This implies that the kind of self-reliance displayed by the participants in this study is likely different from that borrowed from the Western individualist value (63), but are based on the intent of not wanting to create a “burden to their children,” having to “accept the fact that they cannot rely on the younger generation” [(90), p. 7]. Their endorsement of Confucian values of “diligence” and “endurance of hardships” [(92): 49 (93): 2] appears to become their coping resources for the difficulties in life. In this sense, some elders might likely be in need of urgent support even though they do not perceive themselves as in need of help from others or voice such needs. An implication is that it is important to take note of cultural differences and assess the needs of the elderly thoroughly, in both an objective and subjective sense, so as to provide appropriate support for them.

As aforementioned, the results of this study supported the analysis of the life experiences and conditions of the elderly in social isolation using the social systems perspective. From the social systems perspective, there is a “concern for the progressive accommodation between a growing human organism and its immediate environment, and the way in which this relationship is mediated by forces emanating from remote regions in the larger physical and social milieu” [(75): 13]. In this sense, to fully address the needs of these elderly, multi-level intervention and service support are needed. For intervention at the micro level, owing to the elderly’s intensive need for psychological support and care, outreach services, home visits, counseling, and telephone befriending services can be implemented to deal with the individual needs of the elderly and alleviate their sense of isolation and loneliness (94). For the mezzo level of the intervention, it is important for the social service agencies to help the elderly build and strengthen their social support networks in terms of mutual support groups among the elderly and, particularly, family intervention for reducing family conflict and enhancing their family relationships so as to further enhance their psychological well-being. Besides, as engagement in meaningful activities and understanding that they “mean something for others” [(95): 34] are important domains of quality of life, it is important to organize more leisure activities for them and other activities which help dig out their strengths and resources (e.g., voluntary work and interest classes) and help them actualize their potentials in the community as a way to build connections with the community. Providing them with roles in the community and society and contributing helps them feel acknowledged and valued, thus enhancing their sense of self-worth, meaning in life, and empowerment (95, 96). Regarding macro-level interventions, it is important to work on removing welfare stigma in the local culture to encourage the elderly to receive welfare support and enhance their well-being (97). Instead of over-encouraging self-reliance, it is important to normalize the value of mutual aid in society to create an inclusive environment for the elderly. In addition, regarding welfare policy, it is important to advocate a comprehensive retirement protection scheme for the elderly (98), so as to secure their financial support and lead a better fulfilling life.

## 6. Limitations of this study

One important limitation of this study is its small sample size, which cannot fully reflect the phenomenon of social isolation among the elderly in Hong Kong. In the future, similar studies should be conducted with a larger sample. In addition, the participants of this study were mostly women, which would likely exclude the life conditions and situations of the male elderly in social isolation. In the future, similar studies can be conducted with more male participants to generate corresponding practical implications that can fit the needs of different genders. Furthermore, the participants in this study had a low socio-economic status, which caused them to be more vulnerable to social isolation. In the future, other groups of elderly in social isolation can be studied, such as those who engage in active social withdrawal in order to enrich the understanding of these elderly.

## 7. Conclusion

To conclude, the life situations of the elderly in social isolation are affected by multiple levels of factors, which are interwoven, affecting one another. The results reveal that the participants experience social isolation as well as a low sense of satisfaction and self-worth due to their experience of lack, loss, and grief in life, in terms of family, friends, jobs or social roles, and physical ability. This level of well-being reflects their adaptation to the developmental stage of the elderly. Based on Erikson’s (99) psychosocial stages, the developmental task for the eighth stage of ego integrity vs despair normally commenced at the age of 65 and is evident in the elderly’s reflection upon their lives. A successful resolution of this stage is when the elderly “accept what has gone before as inevitable and satisfying,” which results in “integrity,” while an unsuccessful resolution is marked by the elderly’s feeling that “his or her life has been a failure” (100). In this sense, the situations and experiences of the participants manifest the unsuccessful resolution of this stage. Therefore, an implication is that rather than focusing on practical support to fulfill their daily needs (e.g., money and materials), it is more important to provide a range of support services for them that can enhance their adaptation to their developmental stage through the building of social networks and sense of efficacy, so as to enhance their psychological well-being.

## Data availability statement

The original contributions presented in the study are included in the article/supplementary material, further inquiries can be directed to the corresponding author.

## Ethics statement

The studies involving human participants were reviewed and approved by the Ethics Research Committee of City University of Hong Kong. The patients/participants provided their written informed consent to participate in this study.

## Author contributions

All authors listed have made a substantial, direct, and intellectual contribution to the work, and approved it for publication.

## Funding

This study was funded by Karen’s Fund - Simon K. Y. Lee Foundation.

## Conflict of interest

The authors declare that the research was conducted in the absence of any commercial or financial relationships that could be construed as a potential conflict of interest.

## Publisher’s note

All claims expressed in this article are solely those of the authors and do not necessarily represent those of their affiliated organizations, or those of the publisher, the editors and the reviewers. Any product that may be evaluated in this article, or claim that may be made by its manufacturer, is not guaranteed or endorsed by the publisher.

## References

[1] Pruchno R. International aging: spotlighting the spotlights. Gerontologist. (2017) 57:392–5. doi: 10.1093/geront/gnx067

[2] D’cruz M Banerjee D. “An invisible human rights crisis”: the marginalization of older adults during the COVID-19 pandemic – an advocacy review. Psychiatry Res. (2020) 292:113369. doi: 10.1016/j.psychres.2020.113369, PMID: 32795754 PMC7397988

[3] Census and Statistics Department. (2022). 2021 Population Census: Summary Results. Available at: https://www.census2021.gov.hk/doc/pub/21c-summary-results.pdf (Accessed September 01, 2022).

[4] Fischer L. R. Mueller D. B. Cooper P. W.. (1990). Isolated Elderly. Available at: https://files.eric.ed.gov/fulltext/ED330924.pdf (Accessed September 01, 2022).

[5] Holroyd E. Health seeking behaviors and social change: the experience of the Hong Kong Chinese elderly. Qual Health Res. (2002) 12:731–50. doi: 10.1177/10432302012006002, PMID: 12109720

[6] Miao J Wu X. Subjective wellbeing of Chinese elderly: a comparative analysis among Hong Kong, urban China and Taiwan. Ageing Soc. (2021) 41:686–707. doi: 10.1017/S0144686X19001272

[7] Fan R. Which care? Whose responsibility? and why family? A confucian account of long-term care for the elderly. J Med Philos. (2007) 32:495–517. doi: 10.1080/03605310701626331, PMID: 17924274

[8] Raup J Myers JE. The empty nest syndrome: myth or reality? J Couns Dev. (1989) 68:180–3. doi: 10.1002/j.1556-6676.1989.tb01353.x

[9] Census and Statistics Department. (2017). Snapshot of the Hong Kong Population. Available at: https://www.bycensus2016.gov.hk/data/snapshotPDF/Snapshot04.pdf (Accessed September 01, 2022).

[10] Chou K-L Chi I. Comparison between elderly Chinese living alone and those living with others. J Gerontol Soc Work. (2000) 33:51–66. doi: 10.1300/J083v33n04_05

[11] Yeh S-CJ Lo SK. Living alone, social support, and feeling lonely among the elderly. Soc Behav Pers. (2004) 32:129–38. doi: 10.2224/sbp.2004.32.2.129

[12] Dahlberg L Mckee KJ. Correlates of social and emotional loneliness in older people: evidence from an English community study. Aging Ment Health. (2014) 18:504–14. doi: 10.1080/13607863.2013.856863, PMID: 24251626 PMC3979439

[13] Seyfzadeh A Haghighatian M Mohajerani A. Social isolation in the elderly: the neglected issue. Iran J Public Health. (2019) 48:365–6. doi: 10.18502/ijph.v48i2.844, PMID: 31205898 PMC6556198

[14] Wu F Sheng Y. Differences in social isolation between young and old elderly in urban areas of Beijing, China: a cross-sectional study. Int J Nurs Sci. (2020) 7:49–53. doi: 10.1016/j.ijnss.2019.11.003, PMID: 32099859 PMC7031121

[15] Buffel T Rémillard-Boilard S Phillipson C. Social Isolation among Older People in Urban Areas: A Review of the Literature for the Ambition for Ageing Programme in Greater Manchester. Manchester: Manchester Institute for Collaborative Research on Ageing (2015).

[16] Berkman LF Syme L. Social networks, host resistance, and mortality: a nine-year follow-up study of Alameda County residents. Am J Epidemiol. (1979) 109:186–204. doi: 10.1093/oxfordjournals.aje.a112674, PMID: 425958

[17] Seeman TE Berkman LF Blazer DG Rowe JW. Social ties and support and neuroendocrine function: the MacArthur studies of successful aging. Ann Behav Med. (1994) 16:95–106.

[18] Barefoot JC Gronbaek M Jensen G Schnohr P Prescott E. Social network diversity and risks of ischemic heart disease and total mortality: findings from the Copenhagen City heart study. Am J Epidemiol. (2005) 161:960–7. doi: 10.1093/aje/kwi128, PMID: 15870160

[19] Asante S Tuffour G. Social isolation and loneliness in older adults: why proper conceptualization matters. J Ageing Longev. (2022) 2:206–13. doi: 10.3390/jal2030017

[20] Broadhead WE Gehlbach SH DeGruy FV Kaplan BH. Functional versus structural social support and health care utilization in a family medicine outpatient practice. Med Care. (1989) 27:221–33. doi: 10.1097/00005650-198903000-00001, PMID: 2784523

[21] Cacioppo JT Hughes ME Waite LJ Hawkley LC Thisted RA. Loneliness as a specific risk factor for depressive symptoms: cross-sectional and longitudinal analyses. Psychol Aging. (2006) 21:140–51. doi: 10.1037/0882-7974.21.1.140, PMID: 16594799

[22] Hawkley LC Burleson MH Berntson GG Cacioppo JT. Loneliness in everyday life: cardiovascular activity, psychosocial context, and health behaviors. J Pers Soc Psychol. (2003) 85:105–20. doi: 10.1037/0022-3514.85.1.105, PMID: 12872887

[23] Biordi DL Nicholson NR. Social isolation In: Lubkin IM Larsen PD, editors. Chronic Illness: Impact and Intervention. Sudbury, MA: Jones and Bartlett (2013). 85–115.

[24] Meeuwesen L. Life events and social isolation In: Hortulanus R Machielse A Meeuwesen L, editors. Social Isolation in Modern Society. London, England: Routledgepp (2006). 63–80.

[25] Victor A Scambler S Bond J. The Social World of Older People: Understanding Loneliness and Social Isolation in Later Life. New York, NY: McGraw Hill/Open University Press (2009).

[26] Li W Wang Q Yin H Song Y Tu W Wang L. Construction of path analysis model on related factors of social isolation in older people. Psychogeriatrics. (2022) 22:743–56. doi: 10.1111/psyg.12879, PMID: 35859517

[27] Chinese University of Hong Kong. A Qualitative Study on “Hidden Elderly” in Hong Kong. Hong Kong: Central Policy Unit (2008).

[28] Felce D Perry J. Quality of life: its definition and measurement. Res Dev Disabil. (1995) 16:51–74. doi: 10.1016/0891-4222(94)00028-8 7701092

[29] Theofilou P. Quality of life: definition and measurement. Eur J Psychol. (2013) 9:150–62. doi: 10.5964/ejop.v9i1.337

[30] Chin W. P. K.. (2012). “Hidden Elderly: An Analysis from the Individual Perspective and Advice,” in Yautsimmong Mutual Help Programme: A Review on the Effectiveness of the Services for the Hidden Elderly in the Yau Tsim District (Hong Kong: “Strengthening Outreach Work to Assist Hidden Elderly and Needy Elderly” Yau Tsim District Service Unit Joint Conference).

[31] Tsui WT. Supporting hidden elderly: from a social policy perspective In: Chu SC Ho WY Yu KK Lee PY Choi SW, editors. Yautsimmong Mutual Help Programme: A Review on the Effectiveness of the Services for the Hidden Elderly in the Yau Tsim District. Hong Kong: Strengthening Outreach Work to Assist Hidden Elderly and Needy Elderly” Yau Tsim District Service Unit Joint Conference (2012)

[32] Commission on Poverty. (2006). Ad Hoc Group on the Elderly in Poverty, Commission on Poverty Aids Hidden Elderly. Available at: https://www.povertyrelief.gov.hk/archive/2007/b5/pdf/EP_Paper4_2006chi.pdf (Accessed September 01, 2022).

[33] Czaja SJ Moxley JH Rogers WA. Social support, isolation, loneliness, and health among older adults in the PRISM randomized controlled trial. Front Psychol. (2021) 12:728658. doi: 10.3389/fpsyg.2021.728658, PMID: 34675843 PMC8525598

[34] Elderly Health Service. (2023). Social Isolation. Available at: https://www.elderly.gov.hk/english/healthy_ageing/healthy_living/socialisolation.html (Accessed May 01, 2023).

[35] Lai ETC Ho SC Woo J. Social isolation, socioeconomic status, and development of functional impairments in Chinese older adults aged 70 years and over: a cohort study. Aging Clin Exp Res. (2023) 35:155–65. doi: 10.1007/s40520-022-02259-w, PMID: 36273110 PMC9589608

[36] Greaves M Rogers-Clark C. The experience of socially isolated older people in accessing and navigating the health care system. Aust J Adv Nurs. (2009) 27:5–11.

[37] Grundy E. Ageing and vulnerable elderly people: European perspectives. Ageing Soc. (2006) 26:105–34. doi: 10.1017/S0144686X05004484

[38] Waycott J Morgans A Pedell S Ozanne E Vetere F Kulik L. Ethics in evaluating a sociotechnical intervention with socially isolated older adults. Qual Health Res. (2015) 25:1518–28. doi: 10.1177/1049732315570136, PMID: 25646003

[39] Mak Y. F.. (2012). “Understanding the Hidden Elderly’s Needs and Services: From the Perspective of their Individual Psychological and Physical Health” in Yautsimmong Mutual Help Programme: A Review on the Effectiveness of the Services for the Hidden Elderly in the Yau Tsim District (Hong Kong: “Strengthening Outreach Work to Assist Hidden Elderly and Needy Elderly” Yau Tsim District Service Unit Joint Conference).

[40] Chow O. W.. (2012). “Looking at the phenomenon of hidden elderly: their difficulties in adapting to life in old age,” in Yautsimmong Mutual Help Programme: A Review on the Effectiveness of the Services for the Hidden Elderly in the Yau Tsim District (Hong Kong: “Strengthening outreach work to assist hidden elderly and needy elderly” Yau Tsim District Service Unit Joint Conference).

[41] Soulières M Charpentier M. Are older people living alone socially isolated? A qualitative study of their experiences. J Gerontol Soc Work. (2022) 65:664–77. doi: 10.1080/01634372.2021.2019163, PMID: 34986739

[42] Machielse A. Social isolation: formal and informal support In: Hortulanus R Machielse A Meeuwesen L, editors. Social Isolation in Modern Society. London, England: Routledge (2006). 115–36.

[43] Chan AWK Yu DSF Choi KC. Effects of tai chi qigong on psychosocial well-being among hidden elderly, using elderly neighborhood volunteer approach: a pilot randomized controlled trial. Clin Interv Aging. (2017) 12:85–96. doi: 10.2147/CIA.S124604, PMID: 28115837 PMC5221552

[44] Hong Kong Sheng Kung Hui Welfare Council. A Research Report on Services for the Hidden Elderly in Hong Kong. Hong Kong: Hong Kong Sheng Kung Hui Welfare Council (2010).

[45] Hong Kong Special Administrative Region Government News. (2007). Press Releases. Assistance to the Unidentified Elderly [Updated 2007]. Available at: http://www.info.gov.hk/gia/general/200703/28/P200703280174.htm (Accessed May 01, 2023).

[46] Lazarus RS Folkman S. Transactional theory and research on emotions and coping. Eur J Personal. (1987) 1:141–69. doi: 10.1002/per.2410010304

[47] Meeuwesen L. Personal competences and social isolation In: Hortulanus R Machielse A Meeuwesen L, editors. Social Isolation in Modern Society. London, England: Routledge (2006). 81–99.

[48] Meeuwesen L. A typology of social contacts In: Hortulanus R Machielse A Meeuwesen L, editors. Social Isolation in Modern Society. London, England: Routledge (2006). 37–59.

[49] Rokach A Brock H. Coping with loneliness. J Psychol. (1998) 132:107–27. doi: 10.1080/00223989809599269

[50] Åkerlind I Hörnquist JO. Loneliness and alcohol abuse: a review of evidences of an interplay. Soc Sci Med. (1992) 34:405–14. doi: 10.1016/0277-9536(92)90300-F, PMID: 1566121

[51] Machielse A. Sociaal isolement als overlevingsstrategie [Social isolation as coping strategy] In: Jorna T, editor. Mag een Mens Eenzaam Zijn? Studies Naar Existentiële Eenzaamheid en Zingeving [Studies on Existentional Loneliness and Meaning]. Amsterdam, The Netherlands: SWP Publishers (2012). 23–36.

[52] McNeilly DP Burke WJ. Disposable time and disposable income: problem casino gambling behaviour in older adults. J Clin Geropsychol. (2002) 8:75–85. doi: 10.1023/A:1014679507988

[53] Daban F Garcia-Subirats I Porthé V López M de Eyto B Pasarín MI. Improving mental health and wellbeing in elderly people isolated at home due to architectural barriers: a community health intervention. Aten Primaria. (2021) 53:102020. doi: 10.1016/j.aprim.2021.102020, PMID: 33774346 PMC8039551

[54] Fakoya OA McCorry NK Donnelly M. Loneliness and social isolation interventions for older adults: a scoping review of reviews. BMC Public Health. (2020) 20:129. doi: 10.1186/s12889-020-8251-6, PMID: 32054474 PMC7020371

[55] Cohen S Gottlieb BH Underwood LG. Social relationships and health In: Cohen S Underwood LG Gottlieb BH, editors. Measuring and Intervening in Social Support. New York: Oxford University Press (2000). 3–25.

[56] Mueller DP. Social networks: a promising direction for research on the relationship of the social environment to psychiatric disorder. Soc Sci Med. (1980) 14A:147–61. 10.1016/0160-7979(80)90028-4 7209614

[57] Holt-Lunstad J Smith TB Layton JB. Social relationships and mortality risk: a meta-analytic review. PLoS Med. (2010) 7:e1000316. doi: 10.1371/journal.pmed.1000316, PMID: 20668659 PMC2910600

[58] Sandu V. Zólyomi E. Leichsenring K.. (2021). Addressing Loneliness and Social Isolation among Older People in Europe European Centre for Social Welfare Policy and Research: Policy Brief 2021/7. Available at: https://www.age-platform.eu/sites/default/files/AddressingLoneliness%26SocialIsolation-EuropeanCentre-Jul2021.pdf (Accessed May 01, 2023).

[59] Kühner S. Chou K.-L.. (2021). Welfare Stigma Needs to be Addressed to Protect the Incomes of Hong Kong Older Adults. Available at: https://www.ln.edu.hk/f/upload/56682/To%20protect%20the%20incomes%20of%20Hong%20Kong%20older%20adults_Final.pdf (Accessed September 01, 2022).

[60] Zolyomi E.. (2019). Peer Review on “Strategies for Supporting Social Inclusion at Older Age”. Directorate-General for Employment, Social Affairs and Inclusion, European Commission. Available https://ec.europa.eu/social/BlobServlet?docId=21809&langId=en (Accessed May 01, 2023).

[61] Cheng ST Lum T Lam LCW Fung HH. Hong Kong: embracing a fast aging society with limited welfare. Gerontologist. (2013) 53:527–33. doi: 10.1093/geront/gnt017, PMID: 23528290

[62] Wong L.. (2008). Hong Kong’s Welfare Model Reconsidered - What Model?. What Traits? And What Functions? Paper Presented at 2008 EASP 5th Conference on Welfare Reform in East Asia, Taipei, Taiwan.

[63] Treas J Mazumdar S. Older people in America’s immigrant families: dilemmas of dependence, integration, and isolation. J Aging Stud. (2002) 16:243–58. doi: 10.1016/S0890-4065(02)00048-8

[64] Beck U. Risk Society: Towards a Modern Society. London, England: Sage (1992).

[65] Giddens A. Runaway World: How Globalization Is Reshaping our Lives. London, England: Profile Books (2002).

[66] Yeung GTY Fung HH. Social support and life satisfaction among Hong Kong Chinese older adults: family first? Eur J Ageing. (2007) 4:219–27. doi: 10.1007/s10433-007-0065-1, PMID: 28794791 PMC5546368

[67] Cheung C Kam PK. Resiliency in older Hong Kong Chinese: using the grounded theory approach to reveal social and spiritual conditions. J Aging Stud. (2012) 26:355–67. doi: 10.1016/j.jaging.2012.03.004

[68] Lou VWQ Ng JW. Chinese older adults’ resilience to the loneliness of living alone: a qualitative study. Aging Ment Health. (2012) 16:1039–46. doi: 10.1080/13607863.2012.692764, PMID: 22690832

[69] Courtin E Knapp M. Social isolation, loneliness and health in old age: a scoping review. Health Soc Care Community. (2017) 25:799–812. doi: 10.1111/hsc.12311, PMID: 26712585

[70] Hawton A Green C Dickens AP Richards SH Taylor RS Edwards R. The impact of social isolation on the health status and health-related quality of life of older people. Qual Life Res. (2011) 20:57–67. doi: 10.1007/s11136-010-9717-2 20658322

[71] Lim LL Kua EH. Living alone, loneliness, and psychological well-being of older persons in Singapore. Curr Gerontol Geriatr Res. (2011) 2011:673181:1–9. doi: 10.1155/2011/673181 21969827 PMC3182578

[72] Tomova L Wang KL Thompson T Matthews GA Takahashi A Tye KM. Acute social isolation evokes midbrain craving responses similar to hunger. Nat Neurosci. (2020) 23:1597–605. doi: 10.1038/s41593-020-00742-z, PMID: 33230328 PMC8580014

[73] Andersson L. Loneliness research and interventions: a review of the literature. Aging Ment Health. (1998) 2:264–74. doi: 10.1080/13607869856506

[74] Yang K Victor C. The prevalence of and risk factors for loneliness among older people in China. Ageing Soc. (2008) 28:305–27. doi: 10.1017/S0144686X07006848

[75] Bronfenbrenner U. The Ecology of Human Development: Experiments in Nature and Design. Cambridge, MA: Harvard University Press (1979).

[76] Bronfenbrenner U. Ecological systems theory In: Vasta R, editor. Annals of Child Development. Six Theories of Child Development: Revised Formulations and Current Issues. London: Jessica Kingsley (1992). 187–249.

[77] Forder A. Social work and system theory. Br J Soc Work. (1976) 6:23–42.

[78] Luhmann N. Introduction to Systems Theory. Cambridge, MA: Polity Press (2013).

[79] Dunst CJ Vance SD Cooper CS. A social systems perspective of adolescent pregnancy: determinants of parent-child behavior. Infant. Ment. Health. J. (1986) 7:34–48.

[80] Bronfenbrenner U. Ecological models of human development In: Gauvain M Cole M, editors. Readings on the Development of Children. New York: Freeman (1993). 37–43.

[81] Hong JS Espelage DL. A review of research on bullying and peer victimization in school: an ecological system analysis. Aggress Violent Behav. (2012) 17:311–22. doi: 10.1016/j.avb.2012.03.003

[82] Hong Kong Society for the Aged. Hong Kong Island District: A Research on the Needs of the Hidden Elderly. Hong Kong: Hong Kong Society for the Aged (2009).

[83] Overcash JA. Narrative research: a review of methodology and relevance to clinical practice. Crit Rev Oncol Hematol. (2003) 48:179–84. doi: 10.1016/j.critrevonc.2003.04.006 14607381

[84] Ntinda K. Narrative research In: Liamputtong P, editor. Handbook of Research Methods in Health Social Sciences. New York: Springer (2018). 1–12.

[85] Brickman L Rog DJ. Handbook of Applied Social Research Methods. Thousand Oaks, CA: Sage (1998).

[86] Elbow P. Embracing Contraries: Explorations in Teaching and Learning. New York, NY: Oxford University Press (1986).

[87] Elçi A Devran BC. A narrative research approach: the experiences of social media support in higher education In: Zaphiris P and AIoannou, editors. Learning and Collaboration Technologies: Designing and Developing Novel Learning Experiences. LCT 2014. Lecture Notes in Computer Science (Vol 8523). Cham: Springer (2014). 36–42.

[88] Braun V Clarke V. Using thematic analysis in psychology. Qual Res Psychol. (2006) 3:77–101. doi: 10.1191/1478088706qp063oa

[89] Besley T Coate S. Understanding welfare stigma: taxpayer resentment and statistical discrimination. J Public Econ. (1992) 48:165–83. doi: 10.1016/0047-2727(92)90025-B

[90] Du Q Gong N Hu Q Chen G Xie J Luo L. Why do older adults living alone in cities cease seeking assistance? A qualitative study in China. BMC Geriatr. (2022) 22:540. doi: 10.1186/s12877-022-03217-x, PMID: 35768784 PMC9241305

[91] Chow N. Lum T.. (2008). Trends in Family Attitudes and Values in Hong Kong. Final Report Submitted to Central Policy Unit Hong Kong SAR Government. Available at: https://www.researchgate.net/profile/Terry-Y-Lum-2/publication/237336770_Trends_in_Family_Attitudes_and_Values_in_Hong_Kong/links/542a90b60cf277d58e8744d8/Trends-in-Family-Attitudes-and-Values-in-Hong-Kong.pdf (Accessed September 01, 2022).

[92] Li J. Cultural Foundations of Learning: East and West. Cambridge, UK: Cambridge University Press (2012).

[93] Wang CL. Resurgence of Confucian education in contemporary China: parental involvement, moral anxiety, and the pedagogy of memorization. J Moral Educ. (2022):1–18. doi: 10.1080/03057240.2022.2066639

[94] Chen YR Schulz PJ. The effect of information communication technology interventions on reducing social isolation in the elderly: a systematic review. J Med Internet Res. (2016) 18:e18. doi: 10.2196/jmir.4596, PMID: 26822073 PMC4751336

[95] van Leeuwen KM van Loon MS van Nes FA Bosmans JE de Vet HCW Ket JCF. What does quality of life mean to older adults? A thematic synthesis. PLoS One. (2019) 14:e0213263. doi: 10.1371/journal.pone.0213263, PMID: 30849098 PMC6407786

[96] Baumann D Ruch W Margelisch K Gander F Wagner L. Character strengths and life satisfaction in later life: an analysis of different living conditions. Appl Res Qual Life. (2020) 15:329–47. doi: 10.1007/s11482-018-9689-x

[97] Han H Gao Q. Does welfare participation improve life satisfaction? Evidence from panel data in rural China. J Happiness Stud. (2020) 21:1795–822. doi: 10.1007/s10902-019-00157-z

[98] Yu A. The failure of the welfare ideology and system in Hong Kong: a historical perspective. Hum Affairs. (2021) 31:99–108. doi: 10.1515/humaff-2021-0009

[99] Erikson EH. Childhood and Society. 2nd ed. New York: Norton (1963).

[100] Brown C Lowis MJ. Psychosocial development in the elderly: an investigation into Erikson’s ninth stage. J Aging Stud. (2003) 17:415–26. doi: 10.1016/S0890-4065(03)00061-6

